# Minimal Change Disease in a Young Adult With Neurofibromatosis Type 1

**DOI:** 10.7759/cureus.85466

**Published:** 2025-06-06

**Authors:** Patricia B Clissa, Cecilia Maria Lima da Costa, Sabri S Sanabani

**Affiliations:** 1 Immunopathology Laboratory, Butantan Institute, São Paulo, BRA; 2 Pediatric Oncology, A.C.Camargo Cancer Center, São Paulo, BRA; 3 Laboratory of Medical Investigation LIM03, Clinics Hospital, Faculty of Medicine, University of São Paulo, São Paulo, BRA

**Keywords:** corticosteroids, minimal change disease (mcd), neurofibromatosis type 1 (nf1), podocyte injury, proteinuria

## Abstract

Neurofibromatosis type 1 (NF1) is an autosomal dominant disorder characterized by café-au-lait macules, neurofibromas, and a predisposition to various tumors. While the disease is primarily associated with neural and dermatologic manifestations, renal involvement is extremely rare. We present the case of a 20-year-old woman with NF1 who developed steroid-sensitive minimal change disease (MCD) confirmed by renal biopsy. MRI findings revealed plexiform neurofibromas involving the cervical nerve roots and the right temporal region. The patient was started on prednisolone 40 mg/day, achieving remission with normalized proteinuria, but relapsed with edema and proteinuria (7.74 g/L) during tapering. Prednisone was re-initiated at 30 mg/day, tapered to 2.5 mg every other day, and she remains in clinical and biochemical remission while continuing this low dose, with plans to discontinue after one month if remission persists. As researchers, the authors aim to report this rare association to advance scientific understanding of NF1-related renal pathology. This case underscores the importance of considering renal pathology in NF1 patients with nephrotic syndrome and raises the possibility of a biologically plausible link between NF1-related dysregulation of the Ras signaling pathway and podocyte injury as observed in MCD.

## Introduction

Neurofibromatosis type 1 (NF1) is a multisystem genetic disorder with autosomal dominant inheritance, caused by mutations in the NF1 gene on chromosome 17q11.2. This gene encodes neurofibromin, a tumor suppressor that acts as a GTPase-activating protein (GAP), downregulating the Ras/MAPK signaling pathway, which governs cell proliferation and differentiation [1]. Minimal change disease (MCD) is one of the main causes of nephrotic syndrome, particularly in children, but also affects adults. It is essentially a podocytopathy characterized by diffuse effacement of the podocyte foot processes visible by electron microscopy, whereas light microscopy often shows normal-looking glomeruli. The prevailing hypothesis for idiopathic MCD points to a dysregulation of the immune system, probably related to T-cell dysfunction and circulating permeability factors that target podocytes and lead to increased glomerular permeability [2,3]. NF1 affects about 1 in 3,000 people worldwide [4], while MCD is responsible for up to 80%-90% of nephrotic syndrome in children and 10%-15% in adults [5]. While NF1 is primarily known for its cutaneous and neurologic manifestations, renal involvement is uncommon and often underrecognized [6]. Among renal complications, renovascular hypertension from renal artery stenosis is most frequently reported. However, various glomerular diseases including membranous nephropathy, focal segmental glomerulosclerosis, IgA nephropathy, and MCD have also been documented in isolated case reports [6-8]. The pathophysiological basis of glomerular disease in NF1 remains elusive. However, the loss of neurofibromin function in NF1 leads to hyperactivation of the Ras/MAPK signaling pathway [9]. Interestingly, the same signaling pathway together with its downstream effectors such as the mammalian target of rapamycin (mTOR) is crucial for podocyte homeostasis, and its dysregulation has been linked to podocyte injury and the development of proteinuric kidney disease [10,11]. Mechanistic studies suggest that neurofibromin deficiency, which causes hyperactivation of the Ras signaling pathway, could potentially disrupt podocyte function and homeostasis via these downstream signaling pathways [10]. This potential mechanistic overlap between NF1 pathophysiology and the signaling pathways involved in podocyte injury is the reason for investigating the association presented in this case report. Given the rarity of significant glomerular disease in NF1, it is of clinical and scientific interest to understand any potential association, particularly if it is biologically plausible.

## Case presentation

**Figure 1:**
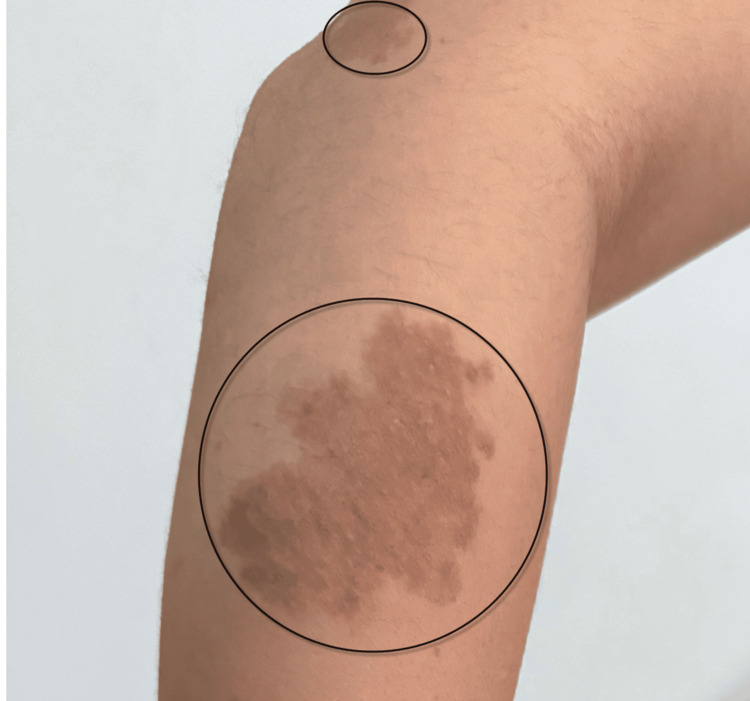
Clinical manifestations of NF1 highlighting café-au-lait macules. A conspicuous brownish mole with underlying fibroma is seen on the right forearm. NF1: neurofibromatosis type 1.

The patient is a 21-year-old woman, born in 2004, with a clinical diagnosis of NF1, representing a new pathogenic variant in the family. Clinical manifestations from birth included multiple café-au-lait macules and pigmentary skin lesions, as shown on the right forearm (Figure 1). A fibroma underlying a brownish mole was observed on the right forearm. Axillary and inguinal freckling were noted on clinical examination but are not depicted in the provided image.

**Figure 2:**
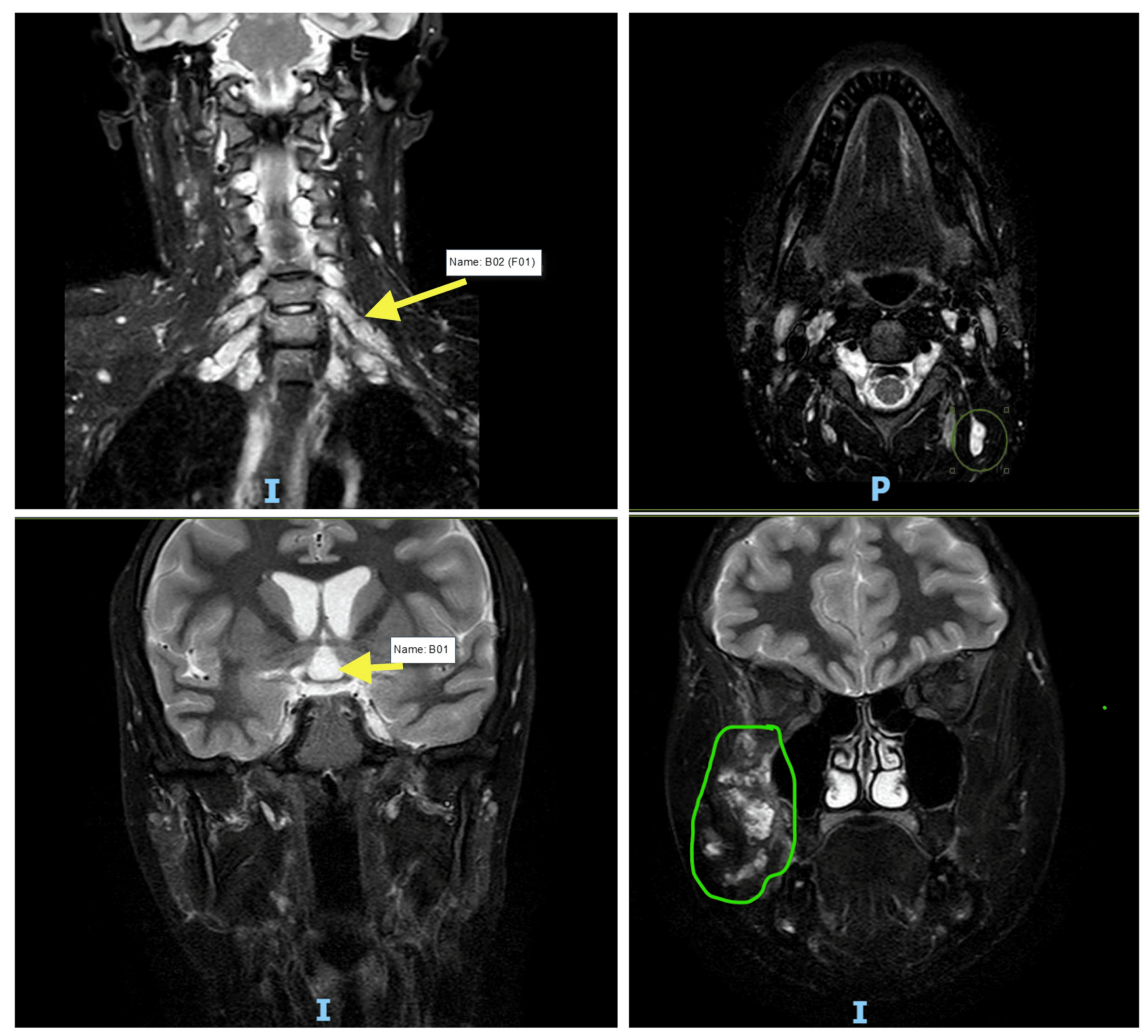
MRI of the patient's cervical and cranial plexiform neurofibromas. MRI images show plexiform neurofibromas involving the cervical nerve roots (C2-C3, C3-C4, C4-C5) and the brachial plexus. The lower images illustrate a lesion in the right temporal and infratemporal regions. The MRI scans are labeled with "I" (inferior) and "P" (posterior), and the green circles highlight the lesions.

The last follow-up MRI images on February 2025 (showing the cranium only) are derived from a whole-body MRI that revealed plexiform neurofibromas involving the cervical nerve roots (C2-C3, C3-C4, C4-C5) and the brachial plexus. A large lesion is seen in the right temporal and infratemporal regions, probably due to a plexiform neurofibroma (Figure 2). Multiple bilateral neurofibromas along several nerve roots throughout the spine and peripheral nerves, consistent with the spinal form of NF1, were identified in the full MRI assessment.

Molecular analysis was conducted using next-generation sequencing to investigate the presence of potentially pathogenic variants. A heterozygous pathogenic variant was identified in the NF1 gene (neurofibromin 1, OMIM 613113), located on chromosome 17 (Chr17:31,357,091 G>A), corresponding to c.7869+1G>A (ENST00000358273), confirming the molecular diagnosis of NF1.

The patient is the first child of a non-consanguineous couple and was born at term with a birth weight of 3.8 kg. At age five, ophthalmologic evaluation revealed myopia, and she was diagnosed with NF1-associated scoliosis and short stature. Scoliosis was managed conservatively with physiotherapy and her development was otherwise normal.

At age 19, she presented with a one-week history of facial and bilateral lower limb edema. On examination, she was afebrile and hemodynamically stable, with a blood pressure of 100/70 mmHg, a heart rate of 80 bpm, and a normal respiratory rate. Her weight had increased to 58 kg from a baseline of approximately 50 kg. Physical examination revealed pitting edema involving the face and lower limbs. Cardiopulmonary and abdominal examinations were unremarkable, and there was no evidence of hepatosplenomegaly or lymphadenopathy.

Initial laboratory work-up revealed significant proteinuria (4.23 g/L) with a low urine creatinine concentration (0.25 g/L), suggesting nephrotic-range protein loss. Serum creatinine was 0.66 mg/dL, albumin was markedly reduced at 2.0 g/dL, and total cholesterol was elevated at 549 mg/dL. Hemoglobin level was within normal limits at 15.5 g/dL (Table 1). Serologic testing for hepatitis B and C, HIV, antinuclear antibody (ANA), and antineutrophil cytoplasmic antibody (ANCA) were all negative.

**Table 1:** Laboratory findings of the patient ANA: antinuclear antibody, ANCA: antineutrophil cytoplasmic antibody.

Laboratory test	Result	Normal range
Serum creatinine	0.66 mg/dL	0.6-1.2 mg/dL
Blood urea nitrogen (BUN)	12 mg/dL	7-20 mg/dL
Serum albumin	2.0 g/dL	3.5-5.0 g/dL
Total protein	5.3 g/dL	6.0-8.3 g/dL
Cholesterol	549 mg/dL	<200 mg/dL
Hemoglobin	15.5 g/dL	12.0-16.0 g/dL (female)
Proteinuria (initial)	4.23 g/L	<0.15 g/L
Proteinuria (relapse)	7.74 g/dL	<0.15 g/dL
Urine creatinine	0.25 g/L	0.5-2.0 g/L
IgM (immunofluorescence)	Mild granular (++)	Negative
Kappa light chain	Positive (+)	Negative
Lambda light chain	Positive (+)	Negative
Hepatitis B and C, HIV, ANA, ANCA	Negative	Negative

**Figure 3:**
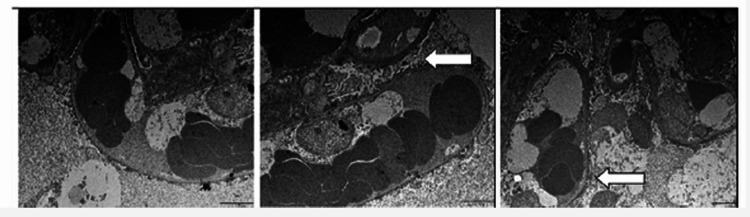
Ultrastructural changes in renal biopsy of our NF1 patient with minimal change disease. Electron microscopy of the renal biopsy shows diffuse effacement of the podocyte foot processes (arrows) and a microvillous transformation of the apical cytoplasm. No electron-dense immune deposits are visible. NF1: neurofibromatosis type 1.

A percutaneous renal biopsy revealed 13 glomeruli with preserved cellularity and normal capillary loops on light microscopy, along with minimal interstitial fibrosis and a small focus of tubular atrophy. The arterioles were within normal limits. Direct immunofluorescence showed mild granular mesangial deposits of IgM (++) and the light chains kappa (+) and lambda (+). Electron microscopy showed diffuse effacement of podocyte foot processes, cytoplasmic degeneration of podocytes with microvillous transformation, and no electron-dense immunodeposits (Figure 3). These findings are consistent with diffuse podocyte damage and are compatible with MCD. Only electron microscopic images are provided in this report.

The patient was initially started on prednisolone 40 mg/day for two weeks, followed by a taper of 10 mg every two weeks down to 20 mg/day. During this period, proteinuria normalized. The tapering continued at a slower rate by 5 mg every two weeks until she reached 15 mg/day. However, she subsequently experienced a relapse, presenting again with edema. At that time, laboratory testing revealed significant proteinuria of 7.74 g/dL.

Prednisone was re-initiated at 30 mg/day for one month, followed by a gradual taper of 5 mg/month down to 5 mg/day, then 2.5 mg every other day. At the time of writing, the patient remained in clinical and biochemical remission. If remission is sustained, corticosteroids will be discontinued after one additional month. In the event of future relapses or steroid dependence, the clinical team plans to consider steroid-sparing agents such as calcineurin inhibitors or mycophenolate mofetil in accordance with the current KDIGO guidelines for the treatment of MCD.

## Discussion

Our case supports the emerging association between NF1 and glomerular disease. The diagnosis of MCD was confirmed histologically, showing characteristic findings of normal-appearing glomeruli on light microscopy and the absence of immune deposits, with no features suggestive of other glomerulopathies. The clinical presentation of nephrotic syndrome and initial responsiveness to corticosteroids is consistent with the natural history of MCD. Although mesangial IgM and light chain deposits were observed on immunofluorescence, these are often considered nonspecific findings in MCD and do not exclude the diagnosis in the absence of mesangial proliferation or electron-dense deposits [12].

The podocytopathy observed by electron microscopy, including widespread foot process effacement and cytoplasmic microvillous transformation, supports the diagnosis of MCD and reflects direct structural injury to podocytes. This aligns with theories proposing that alterations in signaling pathways regulated by neurofibromin may impact podocyte cytoskeletal stability [12]. The NF1 gene product, neurofibromin, plays a crucial role in the downregulation of the Ras/MAPK signaling cascade. Loss of neurofibromin function in NF1 leads to constitutive activation of this signaling pathway [1]. This is significant because Ras signaling and its downstream effectors, including the mTOR pathway, are intimately involved in the regulation of podocyte cell growth, differentiation, cytoskeletal organization, and survival [10,11]. A recent study by Das et al. [13] demonstrated that activation of the TGF-β-ERK1/2-mTORC1 axis plays a key role in promoting podocyte injury and proteinuria in experimental models of glomerular disease, highlighting the pathogenic relevance of these signaling pathways in glomerular permeability and sclerosis. In the context of NF1, where the loss of neurofibromin results in constitutive Ras activation, this may lower the threshold for podocyte injury and contribute to the development of minimal change disease. Dysregulation of these signaling pathways, as occurs systemically in NF1, could therefore increase the susceptibility of podocytes to injury or contribute directly to their dysfunction, leading to increased glomerular permeability, which is a hallmark of MCD. The absence of systemic autoimmune markers and negative viral serologies in our patient strengthens the likelihood of exploring a mechanistic, NF1-related contribution.

Hyun et al. [8] have hypothesized that NF1-related nephrotic syndrome may occur through molecular interactions involving podocyte dysfunction. While specific binding between neurofibromin and glomerular filtration components like syndecan-2 remains speculative, the NF1 gene product has been implicated in maintaining cellular structural integrity and regulating Ras-mediated signaling cascades, as discussed. The clinical and pathologic findings in our patient, including the relatively uncomplicated remission under steroid therapy, are characteristic of idiopathic MCD. Remarkably, our patient achieved remission under corticosteroids despite the constant neurofibromin deficiency and the resulting dysregulation of the Ras signaling pathway in NF1 patients. This suggests that NF1-associated Ras dysregulation does not necessarily alter the baseline steroid sensitivity of MCD (typically suggesting an immune-mediated process), but may act as a predisposing factor that lowers the threshold for podocyte damage by other triggers or perhaps subtly modulates the immune response itself. The fact that MCD has developed suggests a "second hit" or a particular confluence of factors related to the underlying NF1-related overactivity of the Ras signaling pathway.

Interestingly, Hyun et al. [8] also reported complete remission of nephrotic syndrome after surgical resection of a plexiform neurofibroma in an NF1 patient, suggesting a possible paraneoplastic or immunologic mechanism linking NF1-associated tumors to glomerular injury. Our patient is only the second case of MCD associated with NF1 and, more importantly, the first case described in which MCD developed without a concomitantly identified tumor that could be directly implicated in a paraneoplastic mechanism for nephrotic syndrome. The favorable response to corticosteroid therapy supports a steroid-sensitive etiology rather than a structural or tumor-driven mechanism directly causing irreversible glomerular damage in our case. While a coincidental coexistence of NF1 and idiopathic MCD cannot be definitively ruled out, especially given that MCD is the most common cause of nephrotic syndrome in certain age groups, the biological plausibility arising from the common dysregulation of the Ras/mTOR signaling pathway warrants careful consideration of a possible association. The rarity of significant renal involvement in NF1 means that any case of glomerular disease, particularly one such as MCD with potential links to the core molecular defect of NF1, is important to document.

Pediatric studies further support broader renal dysfunction in NF1. Celik et al. [14] reported higher rates of hypertension, tubular dysfunction, and reduced glomerular filtration rate in NF1 patients compared to healthy controls, raising concerns about underappreciated renal involvement even in the absence of overt nephrotic syndrome.

While MCD typically presents in children, it is increasingly recognized in adults, where its course may be more variable and prone to relapses [15]. The patient's initial response to corticosteroids followed by relapse and subsequent remission after a revised tapering regimen aligns with the known behavior of steroid-sensitive nephrotic syndrome in adults [16]. Although idiopathic MCD remains the most common form, secondary MCD must be considered in contexts involving systemic disease, genetic disorders, or neoplasms.

This case raises the possibility that MCD in NF1 patients may reflect a distinct disease phenotype or represent a broader spectrum of NF1-related renal involvement. Whether neurofibromin deficiency creates a predisposition to podocyte injury via Ras-mTOR pathway dysregulation or other intracellular mechanisms warrants further exploration. Given the clinical rarity, future reporting of similar cases, along with molecular and histopathologic correlation, will be essential to understanding the nature of glomerular disease in NF1.

Altogether, while causality between NF1 and MCD remains unproven, the accumulating case reports and plausible molecular pathways suggest that this may be more than a coincidental occurrence. Reporting such cases can catalyze more systematic studies to better characterize renal risks in NF1 populations and potentially lead to early screening or tailored monitoring protocols.

## Conclusions

This case describes a young adult female with NF1 who developed biopsy-confirmed MCD, a rarely reported renal manifestation in this population. The temporal association, steroid responsiveness, and absence of alternative etiologies highlight the need to consider glomerular disease in the differential diagnosis when NF1 patients present with edema or proteinuria. Although the pathogenic link remains speculative, molecular insights into neurofibromin function provide a rationale for further investigation. Increased awareness and more extensive cohort studies are necessary to elucidate the true prevalence and pathogenesis of glomerular diseases in NF1.
